# The Neuronal Calcium Sensor Protein Acrocalcin: A Potential Target of Calmodulin Regulation during Development in the Coral *Acropora millepora*

**DOI:** 10.1371/journal.pone.0051689

**Published:** 2012-12-17

**Authors:** Alejandro Reyes-Bermudez, David J. Miller, Susanne Sprungala

**Affiliations:** 1 ARC Centre of Excellence for Coral Reef Studies and School of Pharmacy and Molecular Sciences, James Cook University, Townsville, Queensland, Australia; 2 Okinawa Institute of Science and Technology, Okinawa, Japan; Russian Academy of Sciences, Institute for Biological Instrumentation, Russian Federation

## Abstract

To understand the calcium-mediated signalling pathways underlying settlement and metamorphosis in the Scleractinian coral *Acropora millepora,* a predicted protein set derived from larval cDNAs was scanned for the presence of EF-hand domains (Pfam Id: PF00036). This approach led to the identification of a canonical calmodulin (AmCaM) protein and an uncharacterised member of the Neuronal Calcium Sensor (NCS) family of proteins known here as Acrocalcin (AmAC). While AmCaM transcripts were present throughout development, AmAC transcripts were not detected prior to gastrulation, after which relatively constant mRNA levels were detected until metamorphosis and settlement. The AmAC protein contains an internal CaM-binding site and was shown to interact *in vitro* with AmCaM. These results are consistent with the idea that AmAC is a target of AmCaM *in vivo*, suggesting that this interaction may regulate calcium-dependent processes during the development of *Acropora millepora*.

## Introduction

Scleractinian corals play important ecological roles, as they are responsible for the underlying framework of coral reefs, one of the most productive ecosystems on earth [1], [2]. However, the molecular mechanisms underlying many aspects of their biology, including symbiosis, calcification and regeneration, are still poorly understood. Calcium metabolism and homeostasis are of particular interest in corals in the context of calcification. Recent microarray studies [3], [4], [5], [6] suggest that calcium-dependent signalling pathways may regulate metamorphosis, symbiosis and skeleton deposition in scleractinian corals. Consistent with this, clear counterparts of many of the molecules known to play key roles in calcium signalling and homeostasis in vertebrates are present in *Acropora* [7], [8]. However, surprisingly little is known about either calcium metabolism or calcium-dependent signalling pathways in corals.

Eukaryotes use changes in intracellular calcium concentration to regulate a diverse variety of cellular signalling pathways [9], [10]. Calcium signalling is regulated by calcium itself via calcium-modulating proteins, which are involved in all aspects of cell function [11]. The EF-hand family is the most studied group of intracellular calcium-binding proteins able to implement the calcium signal or to buffer its cytosolic concentration [9], [12]. Despite sequence and structural similarity, the responses of these “calcium sensors” to binding of calcium are diverse [13]. Upon calcium binding, this group of molecules typically undergoes topological changes within the EF-hand domain, a helix-loop-helix motif [14], enabling interaction with specific target proteins initiating a signalling cascade that will lead to specific cellular responses [13].

Calmodulin (CaM) is considered the most versatile “calcium sensor” due to its role regulating essential cellular processes such as cell cycle and calcium homeostasis across eukaryotes [15], [16]. CaM sequences are known for several cnidarians [17], [18], [19], [20] including *Hydra magnipapillata*, *Nematostella vectensis* and *Acropora* species [18] and CaM expression is up regulated during metamorphosis in the coral *Montastraea faveolata* [5]. Although CaM has been extensively investigated in the context of regulation of many calcium dependent processes, little is known about its interactions in early diverging metazoans and, as a key regulator of calcium-dependent processes, the identification of CaM targets may shed some light on the control of calcium carbonate deposition in corals as well as other processes such as metamorphosis and symbiont interactions. Furthermore, because Scleractinia represent an early diverging animal phylum, unravelling the roles of calcium-dependent process in corals may contribute to understanding the broader evolutionary history of calcium-dependent cellular pathways.

A number of transcripts encoding putative calcium sensor proteins were identified in the transcriptome of *Acropora millepora* [21], amongst which an uncharacterized NCS protein known here as Acrocalcin (AmAC) emerged as a putative AmCaM target as it contains a predicted CaM-binding site. In this study, we characterized AmCaM and AmAC expression profiles as well as the ability of the AmCaM and AmAC proteins to interact *in vitro.* This interaction may be significant during settlement and metamorphosis.

## Materials and Methods

### Collection of Coral Life History Stage

Early coral life history stages were collected on Magnetic Island (Queensland, Australia, GBRMPA Marine Park Permit G10/33232.1) and maintained in fresh filtered seawater (1 µm) prior to either snap freezing in liquid nitrogen (for RNA extraction) or fixation in 4% formaldehyde (for *in-situ* hybridisation; [22]).

### RNA Extraction and Virtual Northern Blotting

Total RNA was extracted from the following key stages of *Acropora millepora* development: 1) the pre-gastrulation “prawnchip” (PC) stage, 2) late gastrula “sphere” (S), 3) early planula “pear” (Pea), 4) planula larva (Pla) and 5) settled juvenile polyps (Post). RNA extractions were performed using Ambion RNAwiz™ RNA isolation Briefly,1 µg samples of total RNA were used from each sample to synthesise first strand cDNA, 300 ng aliquots of which were subjected to limited PCR amplification using the SMART PCR cDNA synthesis kit as described previously [23]. To generate virtual northern blots, 2 µl aliquots of the resulting double-stranded (stage specific) cDNA samples were loaded into independent wells on agarose gels and transferred to nylon membranes (Hybond-N+, Amersham) by capillary blot transfer and hybridised as described [24]. Because the amount of starting material (RNA) is uniform and subject to PCR within the range where amplification is directly proportional to the number of cycles performed, hybridisation signals detected after virtual northern analysis are directly comparable and reflect levels of transcript present at each developmental stage.

### Generation of Radioactive Probes

Radioactive probes were prepared by random oligonucleotide-primed synthesis (oligolabelling) using α-^32^P dATP (Geneworks). Linear DNA (25 ng) was radioactively labelled using the Megaprime oligolabelling kit (Amersham Biosciences). Membranes were exposed to Phosphorimager screens (Molecular Dynamics) for 5 h.

### Expression of Recombinant Proteins

Complete coding sequences of AmCaM and AmAC were cloned into pGEX-6P (GE-Healthcare) or,pProEX HTb (Invitrogen) respectively, allowing expression in *E. coli* BL21 of fusion proteins carrying either GST- (pGEX-6P) or 6×His (pProEX) tags at their N-termini. To induce expression of the fusion proteins, IPTG was added to cultures of optical density 0.5–0.8 at 600 nm to a final concentration of 1 mM. Three hours after IPTG treatment, cells were harvested by centrifugation at 4000 rpm for 15 min at 4°C. Pellets were suspended in 10 ml aliquots of ice cold PBS and lysed by sonication. Cell debris was pelleted by centrifugation at 10,000 rpm for 5 min and supernatants subjected to affinity chromatography on the appropriate ligand. Using the manufacturers’ recommended protocols.

### Affinity Purification of Recombinant Proteins

To 0.25 ml of either equilibrated 50% Glutathione Sepharose 4B suspension beads (Pharmacia Biotech) or Ni-NTA resin (QIAGEN) recovered supernatants were added and purifications were carried out according to the manufacturers’ protocols. Eluted samples were subjected to standard protein electrophoresis on a 10% acrylamide gel according to [24]. Gels were stained with Coomassie brilliant blue and the sizes of recombinant proteins estimated by comparison with standard commercial protein standards.

### Protein Interaction Experiments Using Affinity Chromatography

Aliquots (0.5 ml) of sonicated soluble fractions of GST-AmCaM and His-AmAC preparations were incubated with 0.25 ml of Ni-NTA resin for one hour at 4°C with shaking in the presence of either 1 mM CaCl_2_ or 5 mM EGTA. Mixtures were then loaded onto a protein purification column and treated with wash and elution buffers containing either 1 mM CaCl_2_ or 5 mM EGTA. Eluted and flow through samples were subjected to standard protein electrophoresis to test for co-localization of GST-AmCaM and His-AmAC proteins within the same fraction.

### Protein Interaction Experiments Using Immunoprecipitation

Aliquots (0.5 ml) of sonicated soluble fractions of GST-AmCaM and His-AmAC preparations were mixed and incubated at 4°C for 1 h with shaking in the presence of either 1 mM CaCl_2_ or 5 mM EGTA. After this time, an aliquot (50 µl) of agarose-conjugated mouse antibody to human calmodulin, raised against amino acids 1–149 of the full length human Calmodulin I (CaM-I; Santa Cruz sc-5537 AC) in a 500 µg/ml stock solution, was added and incubation continued for another 1 h at 4°C with shaking, prior to collection of immunoprecipitates by centrifugation and washing of the pellets (X3) with 0.5 ml aliquots of PBS containing either 1 mM CaCl_2_ or 5 mM EGTA. The resulting immunoprecipitates and supernatants were analysed by protein electrophoresis followed by western blotting [24].

### *In situ* Hybridization

The template for riboprobe production was generated from mixed stage cDNA by PCR using the following primer pair: forward (5′-GCACGAGTGGCACTGTACG) and reverse (5′-TGAAATTCTAGCTCACGGAAAA) and the product cloned into pGEM-T (Promega). Antisense and control sense strand RNA-probes were generated, and *in situ* hybridization performed as previously described [22], with the exception that clearing and photography were carried out as described by [25].

## Results

### Identification and Characterization of EF-hand Proteins

A predicted protein set derived from early stage *A. millepora* cDNA libraries was scanned for the presence of EF-hand domains (Pfam Id: PF00036). Several cDNAs encoding putative calcium sensor proteins were identified; two of these clearly corresponded to widely distributed proteins - a canonical calmodulin (CaM) protein, designated here as AmCaM and an uncharacterised member of the Neuronal Calcium Sensor (NCS) family of proteins known here as Acrocalcin (AmAC).

**Figure 1:**
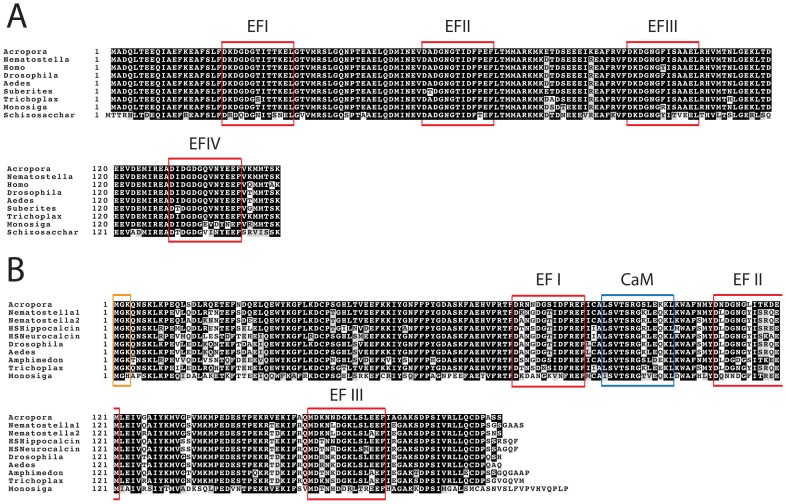
Primary structure of the coral EF-hand proteins. (A) As in the canonical calmodulins of a wide range of other eukaryotes, the AmCaM protein contains four predicted EF-hand motifs, each of which fulfils the criteria for activity. Genbank identifiers for the sequences: *Acropora* Cluster 043479; *Nematostella* XP_00163858.1; *Homo* NP_001734.1; *Drosophila* NP_523710.1; *Aedes* XP_001662431.1; *Suberites* O97341; *Trichoplax* EDV29861.1; *Monosiga* XP_001749021.1; *Schizosaccharomyces* XP_002175972. (B) The coral Acrocalcin (AmAC) protein is a typical member of the NCS-B class, possessing an N-terminal myristoylation site (MGK, orange box), three EF-hand motifs (indicated by red boxes) and a predicted CaM-binding site (blue box). Genbank identifiers for sequences: *Acropora* Cluster 013002; *Nematostella1* XP_001639634.1; *Nematostella2* XP_001639635.1; HS (*Homo sapiens*) hippocalcin NP_002140.2; HS (*Homo sapiens*) neurocalcin NP_114430; *Drosophila* NP_788543.1; *Aedes* XP_001648788.1; *Amphimedon* XP_003386697.1; *Trichoplax* EDV23214.1; *Monosiga* EDQ90181.1.

AmCaM encodes an acidic protein of 149 AA with predicted molecular weight (MW) of ∼17 kDa and isoelectric point (pI) of 4.15. The AmCaM protein (corresponding to Cluster 043479; Fig. 1A) has a high level of similarity with canonical CaM molecules from other species, e.g. 100% identity to sequences from *Acropora muricata* and *A. digitifera* (ACA51013.1, and aug_v2a.01102.t1 respectively), approximately 99% identity with *Nematostella* XP_001638581.1 and 97% identity to the human CaM (NP_001734.1). Interestingly, we were unable to identify a canonical calmodulin in the genome of the sponge *Amphimedon*, although clear orthologs were present in two other sponges, *Suberites domuncula* and *Halicondria okadi*, the former of which is included in the alignment shown as Fig. 1A. The canonical CaM protein is remarkably similar across the eukaryotes, the yeast (*Schizosaccharomyces*) sequence having 73% identity with the coral sequence. AmCaM is typical in terms of structure; the protein contains four EF-hand motifs (each of ∼13 AA), each of which fulfils the criteria for Ca^2+^-binding activity.

The coral NCS protein AmAC (corresponding to Cluster 013002) (Fig. 1B) encodes a protein of 190AA with a predicted MW of ∼22 kDa and predicted pI of 5.03. BlastP significance and identity values against the canonical NCS-B proteins Neurocalcin from *Drosophila* (NP_788543.1, E = 3e^−116^ and 85% identity) and Hippocalcin from the mosquito *Aedes aegypti* (XP_001648788.1, E = 6e^−116^ and 84% identity) identify the AmAC protein as an uncharacterised NCS-B class member [26]. Like some other NCS-B proteins, AmAC contains three EF-hand motifs and an N-terminal myristoylation site (MGK). This modification allows the association of NCS proteins with targets that are usually membrane associated or directs them to specific subcellular compartments in a calcium dependent manner [10], [26].

### After Gastrulation AmCaM and AmAC Genes are Co-expressed during Coral Development

**Figure 2:**
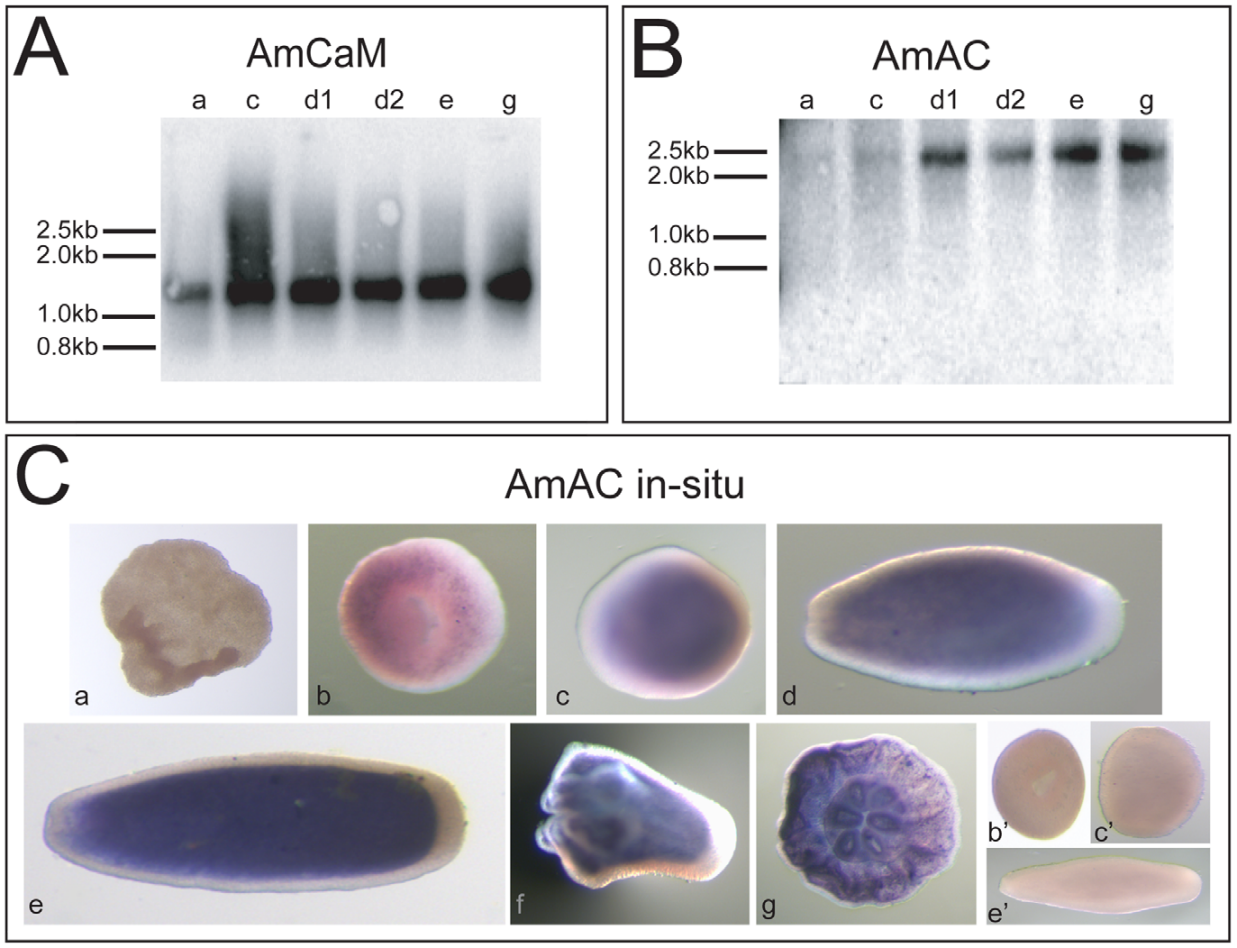
Expression of calmodulin and Acrocalcin during coral development. (A) The canonical calmodulin AmCaM is expressed at a relatively constant level during development as a ∼ 1500 bp transcript. (B) The ∼2500 bp Acrocalcin (AmAC) transcript is present at low levels at the late gastrulation stage, after which levels are relatively constant from the pear stage through to post-settlement. An early and a late pear stage were tested for AmCaM and AmAC transcripts. (C) The faint salt-and-pepper pattern visible at gastrulation reflects AmAC expression in the endoderm (b). A relatively constant expression level was observed from late gastrulation onward, sphere through post-settlement (c–g). No staining was detected in corresponding controls incubated with sense RNA probes (b′, c′, e′). Nucleic acid markers (asterisks). Pre-gastrulation (prawn chip, a). Gastrulation (donut, b). Late gastrula (sphere, c). Early planula (pear, d). Planula (e). Settlement and metamorphosis (f). Settled polyps (g). Control stages (b′, c′, e′), respectively.

Northern blotting revealed that the developmental profiles of the transcripts encoding the two *Acropora* EF-hand proteins differed. Whilst AmCaM transcripts (∼1500 bp) were present at relatively uniform levels across each of the developmental stages examined (prawn chip to post settlement; Fig. 2A), the (∼2500 bp) AmAC transcript was first detected at late gastrulation and was present higher levels from the pear stage through to post settlement (Fig. 2B). In situ hybridization with AmAC probes revealed a faint, salt-and-pepper like pattern of expression in the gastrula (donut) (Fig. 2C: b), expression becoming uniform but restricted to the endoderm from the early planula (sphere) stage through to post-settlement (Fig. 2C, c–g). No staining was detected prior to gastrulation (Fig. 2C, a) or when using the sense probe (Fig. 2C: b′, c′ and e′). In situ hybridization with various probes implied that AmCaM was expressed ubiquitously throughout development (data not shown).

The major developmental stages of embryonic development until early settlement of *Acropora* together with an approximate timeline are summarized in [27]. The embryo at “prawn chip” stage (Figure 2C-a) consists of an irregularly shaped cellular bilayer. During the next stage “donut” (Figure 2C-b and –b′), the morphogenetic movements of gastrulation result in the formation of the two germ layers, ectoderm and endoderm with a cell-free mesoglea between. At about 28–36 hrs the embryo becomes spherical (Figure 2C-c and –c′) with a closing blastopore marking the end point of gastrulation and the embryonic life. At the start of larval life, the larva becomes “pear”-shaped (Figure 2C-d), cilia develop and an oral pore appears at the posterior end, as defined by the direction of swimming. In the later larval stages the planula elongates to a “spindle” (Figure 2C-e and e′) with numerous differentiated cell types [22], [27], [28]. On receipt of appropriate settlement cues, e.g. crustose coralline algae [29], the coral planula attaches to the substrate by the aboral end, contracts along the oral-aboral axis forming a flattened disc that becomes radially subdivided by mesenteries during the process of permanent settlement (Figure 2C-g) and metamorphosis (Figure 2C-f) into a juvenile coral polyp [6], [23], [30]. Associated with metamorphosis from planula to polyp and the start of calcification of the complex species-specific aragonite exoskeleton is dramatic reorganisation of certain tissues [30], [31].

Note that the calmodulin studied here (AmCaM) is clearly distinct from the A61 calmodulin previously reported [23]; the proteins encoded by these loci have only 48% identity. AmCaM and A61 have very different temporal expression patterns, the former being ubiquitously expressed in all stages, whereas in the latter expression was restricted to larval stages (see Fig. 2 in [23]). Given the complexity of early coral development, the requirement for multiple EF-hand proteins with overlapping patterns of expression is consistent with complex roles for calcium in regulating a wide range of processes [10], [16], [32].

### AmCaM and AmAC Proteins Interact *in vitro*

Use of the Calmodulin Target Database [33] allowed the identification of a potential CaM-binding site (13AA) between motifs EFI and EFII in the AmAC protein (Fig. 1B), suggesting that the AmAC and AmCaM proteins might interact in vivo. The AmAC CaM binding motif (LSVTSRGSLEKKL) seems to be a novel structure combining characteristics of canonical IQ and 1-8-14 motifs [34]. Although this site lacks some canonical IQ residues, the terminal residues (KKL) are characteristic of IQ motifs from neurite growth related proteins and the internal RG sequence is commonly associated with the IQ motifs of myosin molecules [34]. Furthermore, the presence of flanking and internal bulky hydrophobic residues such as leucines (L) resembles the structure of 1-8-14 motifs [33] with the difference that the anchoring residues of the calmodulin binding site are in position 1-9-13.

**Figure 3:**
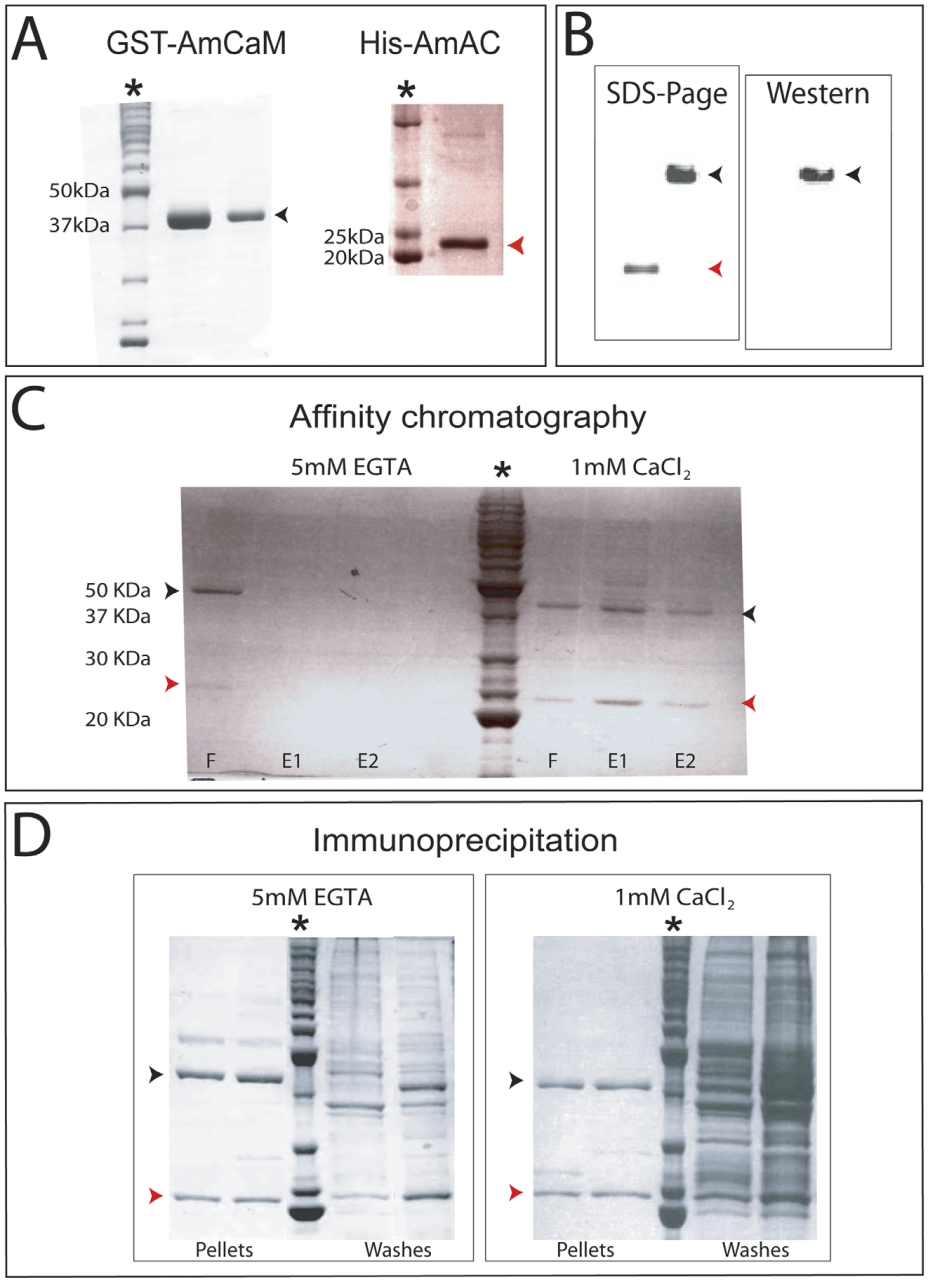
The Acrocalcin and calmodulin proteins interact *in vitro*. (A) Purity of preparations of the recombinant proteins used in the *in vitro* interaction experiments: *Acropora* calmodulin carrying an N-terminal GST-tag (GST-AmCaM; ∼42 KDa) and Acrocalcin bearing an N-terminal poly-His tag (His-AmAC; ∼24 KDa). (B) Polyclonal antibody against human canonical calmodulin specifically recognises recombinant AmCaM (black arrow), whereas recombinant AmAC (red arrow) is not recognised. (C) In the presence of Ca^2+^ (right panel), AmCaM is retained on a Ni-NTA affinity column via its interaction with the recombinant AmAC that attaches to the matrix via the poly(His) tag that it contains, whereas in the absence of Ca^2+^ (left panel) neither protein is retained by the column. (D) Recombinant AmAC is co-precipitated with AmCaM after incubation with antibody against human calmodulin (agarose-conjugated human CaM-I antibody; Santa Cruz) both in the presence (right panel) and absence (left panel) of Ca^2+^. GST-AmCaM (black arrows). His-AmAC (red arrows). Flow through (F), Elution 1 (E1), Elution 2 (E2). Protein markers (asterisks).

Virtual northern blotting experiments (Fig. 2A, B) showed that AmCaM and AmAC are both expressed during late larval development, when metamorphosis and settlement occur and calcification is initiated. As CaM transcript levels are relatively constant through development, the interaction may be regulated by the availability of AmAC [12], [35]. To test the ability of AmCaM to interact with AmAC, affinity chromatography and immunoprecipitation experiments were conducted in the presence of available calcium (1 mM CaCl_2_) or in its absence (5 mM EGTA). For this purpose, recombinant GST-AmCaM (∼42 KDa) and His-AmAC (∼24 KDa) proteins were produced and purified (Fig. 3A).

Affinity chromatography confirmed the interaction of the AmCaM and AmAC proteins predicted on the basis of sequence analysis. After incubating the fusion-proteins together in the presence or absence of calcium, the resulting protein complexes were subjected to chromatography using the His-tag affinity ligand Ni-NTA (Fig. 3C). As can be seen in the right panel of Fig. 3C, in the presence of Ca^2+^, GST-AmCaM was retained on the Ni-NTA affinity column via its interaction with AmAC (which carries the poly(His) affinity tag), as the two proteins are eluted together in E1 and E2. In the absence of Ca^2+^ (left panel of Fig. 3C), neither protein was retained by the column, because the interaction of the affinity (poly-His) tag with the column is prevented in the presence of EGTA.

The observed discrepancies in size of the fusion-proteins (GST-AmCaM and His-AmAC) between the 5 mM EGTA and the 1 mM CaCl_2_ treatments (Fig. 3C), is explained by topological changes experienced in Ca^2+^-binding molecules under denaturing gel electrophoresis. In the presence of calcium, CaM molecules are known to migrate faster on the SDS gels, thus having smaller apparent molecular weights than in the absence of calcium [36], [37] (Fig. 3C, black arrow heads). The same phenomenon was observed during denaturing gel electrophoresis of the calcium-binding protein AmAC (Fig. 3C, red arrow heads).

To test whether the AmAC/AmCaM interaction occurs in the absence of Ca^2+^, the recombinant proteins were incubated together in medium containing 1 mM CaCl_2_ or 5 mM EGTA and then subjected to immunoprecipitation using anti-human calmodulin. The anti-human CaM-I antibody detected specifically the *Acropora* CaM (Fig. 3B). As can be seen in Fig. 3D, AmAC was co-precipitated with AmCaM in both the presence and absence of Ca*2+*, implying that the AmAC/AmCaM interaction occurs in a calcium-independent manner *in vitro*.

## Discussion

In eukaryotes, regulatory EF-hand proteins are the primary mediators of calcium signalling pathways [38], and amongst these, the Neuronal Calcium Sensor (NCS) protein family appear to have a wide variety of roles [39], [40]. Five classes (A-E) of NCS protein are recognized based on sequence similarity [10], [39].

While members of the NCS-A class, including NCS-1 and frequenin [41], [42], are widely distributed across eukaryotes, the coral NCS protein reported here clearly belongs to the NCS-B class, members of which were previously known only from bilaterians [38], [43]. Searching the database revealed the presence of two uncharacterised genes encoding predicted NCS-B proteins (Nv1: XP_001639634 and Nv2: XP_001639635) in the genome of the sea anemone *Nematostella vectensis* genome [44]. Clearly related NCS-B sequences are also present in the sponge *Amphimedon* (XP_003386697.1) and the placozoan *Trichoplax* (EDV23214.1). NCS proteins are also present in the choanoflagellate *Monosiga*, but at this time it is unclear to which class the closest *Monosiga* match (EDQ90181.1) belongs. Thus the NCS-B protein class clearly pre-dates the Bilateria, but whether it predates the Metazoa is unclear.

Despite canonical calmodulins being highly conserved, they participate in a diverse range of biological processes due to the ability to interact with an enormous range of target proteins, many of which are taxonomically restricted [12], [35]. For example, in another coral, *Stylophora pistillata*, CaM is thought to interact with a calcium-ATPase, and this interaction may be relevant to the regulation of skeleton deposition [45]. The presence of clear counterparts of most of the key molecules involved in vertebrate calcium signalling as well as a number of coral-specific calcium sensors (in preparation) suggests that both conserved and coral-specific calcium dependent signalling pathways function during settlement and metamorphosis in *A. millepora.* Given the wide phylogenetic distribution and sequence conservation displayed by both calmodulin and NCS-B proteins, it is surprising that the interaction identified here has not been previously reported, however, we were unable to find precedents in the literature or the String interaction databases [string-db.org] or IntAct [ebi.ac.uk/intact].

The affinity chromatography and immunoprecipitation experiments presented here demonstrate that AmCaM interacts with AmAC *in vitro* in the presence of calcium (Figs. 3C and D)., and immunoprecipitation experiments indicate that this interaction is likely to also occur in the absence of calcium (Figure 3D, left panel).

The temporal expression data (Fig. 2) are consistent with this interaction occurring during late larval development in *Acropora*, although the in situ data indicate that this is more likely to occur in the endoderm than in the aboral ectoderm that is the site of calcification initiation. The fact that the putative CaM binding site is located between two EF-hand domains in the AmAC protein suggests that cooperative coordination may occur between the calcium binding motifs of the two proteins. For some target proteins, CaM coordination is necessary to enable calcium binding or to induce exposure of hydrophobic residues [10], [46], [47], [48].

Most NCS family members are thought to be multifunctional regulators of neuronal cellular processes [39], [49], only NCS-A types being known to function in both neuronal and non-neuronal cell types [39], [50], [51]. Whereas NCS-B proteins have neuronal functions in vertebrates, the broad endodermal expression pattern observed for AmAC (Fig. 3) suggests a non-neuronal role during coral development. Analogy with the vertebrate NCS proteins neurocalcin and hippocalcin suggests possible roles in vesicle or mRNA transport, regulation of cGMP intracellular levels and/or apoptosis [38], [52], [53] in the larval endoderm.

Coral larvae accumulate calcium in endodermal lipid containing vesicles prior to skeleton deposition [54]. During the development of *Acropora* larvae, endodermal tissue functions primarily in the mobilisation of stored lipids for energy metabolism and buoyancy control [31], [55], [56], presumably also releasing calcium from endodermal lipid stores. AmAC might therefore have a regulatory role during the calcium release process that accompanies metamorphosis and skeleton deposition. Clarifying the role of AmAC and the biological relevance of the AmAC/AmCaM interaction will require the development of tools to allow functional analysis of coral genes.

## References

[1] Gunderson L (2007) Ecology: a different route to recovery for coral reefs. Curr Biol 17: R27–28. 17208175 10.1016/j.cub.2006.11.034

[2] Veron J (2000) Corals of the world. Townsville: Australian Institute of Marine Sciences.

[3] DeSalvo MK, Voolstra CR, Sunagawa S, Schwarz JA, Stillman JH, et al (2008) Differential gene expression during thermal stress and bleaching in the Caribbean coral *Montastraea faveolata* . Mol Ecol 17: 3952–3971. 18662230 10.1111/j.1365-294X.2008.03879.x

[4] Grasso LC, Maindonald J, Rudd S, Hayward DC, Saint R, et al (2008) Microarray analysis identifies candidate genes for key roles in coral development. BMC Genomics 9: 540. 19014561 10.1186/1471-2164-9-540 PMC2629781

[5] Reyes-Bermudez A, Desalvo MK, Voolstra CR, Sunagawa S, Szmant AM, et al (2009) Gene expression microarray analysis encompassing metamorphosis and the onset of calcification in the scleractinian coral *Montastraea faveolata* . Marine Genomics 2: 149–159. 21798184 10.1016/j.margen.2009.07.002

[6] Grasso LC, Negri AP, Foret S, Saint R, Hayward DC, et al (2011) The biology of coral metamorphosis: molecular responses of larvae to inducers of settlement and metamorphosis. Dev Biol 353: 411–419. 21338599 10.1016/j.ydbio.2011.02.010

[7] Miller DJ (2011) Coralbase: *Acropora millepora* genome assembly pre-release www.coralbase.org.

[8] Shinzato C, Shoguchi E, Kawashima T, Hamada M, Hisata K, et al (2011) Using the *Acropora digitifera* genome to understand coral responses to environmental change. Nature 476: 320–323. 21785439 10.1038/nature10249

[9] Carafoli E (2002) Calcium signaling: a tale for all seasons. Proc Natl Acad Sci U S A 99: 1115–1122. 11830654 10.1073/pnas.032427999 PMC122154

[10] Haeseleer F, Imanishi Y, Sokal I, Filipek S, Palczewski K (2002) Calcium-binding proteins: intracellular sensors from the calmodulin superfamily. Biochem Biophys Res Comm 290: 615–623. 11785943 10.1006/bbrc.2001.6228

[11] Carafoli E, Genazzani A, Guerini D (1999) Calcium controls the transcription of its own transporters and channels in developing neurons. Biochem Biophys Res Comm 266: 624–632. 10603299 10.1006/bbrc.1999.1879

[12] Bähler M, Rhoads A (2002) Calmodulin signalling via the IQ motif. FEBS Letters 513: 107–113. 11911888 10.1016/s0014-5793(01)03239-2

[13] Bhattacharya S, Bunick CG, Chazin WJ (2004) Target selectivity in EF-hand calcium binding proteins. Biochim Biophys Acta 1742: 69–79. 15590057 10.1016/j.bbamcr.2004.09.002

[14] Gifford JL, Walsh MP, Vogel HJ (2007) Structures and metal-ion-binding properties of the Ca2+-binding helix-loop-helix EF-hand motifs. Biochem J 405: 199–221. 17590154 10.1042/BJ20070255

[15] Cyert MS (2001) Genetic analysis of calmodulin and its targets in Saccharomyces cerevisiae. Ann Rev Genet 35: 647–672. 11700296 10.1146/annurev.genet.35.102401.091302

[16] Carafoli E (2005) Calcium–a universal carrier of biological signals. Delivered on 3 July 2003 at the Special FEBS Meeting in Brussels. FEBS J 272: 1073–1089. 15720383 10.1111/j.1742-4658.2005.04546.x

[17] Jamieson GA, Bronson DD, Schachat FH, Vanaman TC (1980) Structure and function relationships among Calmodulins and tropinin C-like proteins from divergent eukaryotic organisms. Annals NY Acad Sci 356: 1–13. 10.1111/j.1749-6632.1980.tb29593.x 6263143

[18] Morita M, Iguchi A, Takemura A (2009) Roles of calmodulin and calcium/calmodulin-dependent protein kinase in flagellar motility regulation in the coral *Acropora digitifera* . Mar Biotech 11: 118–123. 10.1007/s10126-008-9127-4 18661183

[19] Yuasa HJ, Suzuki T, Yazawa M (2001) Structural organization of lower marine nonvertebrate calmodulin genes. Gene 279: 205–212. 11733145 10.1016/s0378-1119(01)00755-7

[20] Chen IP, Tang CY, Chiou CY, Hsu JH, Wei NV, et al (2009) Comparative analyses of coding and noncoding DNA regions indicate that *Acropora* (Anthozoa: Scleractina) possesses a similar evolutionary tempo of nuclear vs. mitochondrial genomes as in plants. Mar Biotech 11: 141–152. 10.1007/s10126-008-9129-2 18670809

[21] Moya A, Huisman L, Ball EE, Hayward DC, Grasso LC, et al (2012) Whole transcriptome analysis of the coral *Acropora millepora* reveals complex responses to CO_2_-driven acidification during the initiation of calcification. Mol Ecol 21: 2440–2454. 22490231 10.1111/j.1365-294X.2012.05554.x

[22] Hayward DC, Catmull J, Reece-Hoyes JS, Berghammer H, Dodd H, et al (2001) Gene structure and larval expression of cnox-2Am from the coral *Acropora millepora* . Dev Genes Evol 211: 10–19. 11277400 10.1007/s004270000112

[23] Hayward DC, Hetherington S, Behm CA, Grasso LC, Foret S, et al (2011) Differential gene expression at coral settlement and metamorphosis - a subtractive hybridization study. PloS One 6: e26411. 22065994 10.1371/journal.pone.0026411 PMC3204972

[24] Sambrook JF, Fritsch EF, Maniatis T (1989) Molecular Cloning: A laboratory manual. Cold Spring Harbour, New York: Cold Spring Harbour Press.

[25] de Jong DM, Hislop NR, Hayward DC, Reece-Hoyes JS, Pontynen PC, et al (2006) Components of both major axial patterning systems of the Bilateria are differentially expressed along the primary axis of a ‘radiate’ animal, the anthozoan cnidarian *Acropora millepora* . Dev Biol 298: 632–643. 16952346 10.1016/j.ydbio.2006.07.034

[26] Burgoyne RD, O’Callaghan DW, Hasdemir B, Haynes LP, Tepikin AV (2004) Neuronal Ca^2+^-sensor proteins: multitalented regulators of neuronal function. Trends Neurosci 27: 203–209. 15046879 10.1016/j.tins.2004.01.010

[27] Ball EE, Hayward DC, Reece-Hoyes JS, Hislop NR, Samuel G, et al (2002) Coral development: from classical embryology to molecular control. Int J Dev Biol 46: 671–678. 12141456

[28] Ball EE, Hayward DC, Catmull J, Reece-Hoyes JS, Hislop NR, et al. (2000) Molecular control of development in the reef coral, *Acropora millepora* Proc 9^th^ Int Coral Reefs Symp: 395–401.

[29] Morse ANC, Iwao K, Baba M, Shimoike K, Hayashibara T, et al (1996) An ancient chemosensory mechanism brings new life to coral reefs. Biol Bull 191: 149–154. 29220228 10.2307/1542917

[30] Harrison PL, Wallace CC (1990) Reproduction, dispersal and recruitment of Scleractinian corals. In: Dubinsky Z, editor. Coral Reefs. Amsterdam: Elsevier Science Publisher BV 133–207.

[31] Vandermeulen JH (1975) Studies on Coral Reefs.III. Fine Structural Changes of Calicoblast Cells in *Pocillopora damicornis* during Settling and Calcification. Mar Biol 31: 69–77.

[32] Vetter SW, Leclerc E (2003) Novel aspects of calmodulin target recognition and activation. Eur J Biochem 270: 404–414. 12542690 10.1046/j.1432-1033.2003.03414.x

[33] Lab I (2002) Calmodulin Target Database http://calcium.uhnres.utoronto.ca/ctdb/.

[34] Rhoads AR, Friedberg F (1997) Sequence motifs for calmodulin recognition. FASEB J 11: 331–340. 9141499 10.1096/fasebj.11.5.9141499

[35] Benaim G, Villalobo A (2002) Phosphorylation of calmodulin. Functional implications. Eur J Biochem 269: 3619–3631. 12153558 10.1046/j.1432-1033.2002.03038.x

[36] Wallace RW, Tallant EA, Dockter ME, Cheung WY (1982) Calcium binding domains of calmodulin. Sequence of fill as determined with terbium luminescence. J Biol Chem 257: 1845–1854. 6276400

[37] Burgess WH, Jemiolo DK, Kretsinger RH (1980) Interaction of calcium and calmodulin in the presence of sodium dodecyl sulfate. Biochim Biophys Acta 623: 257–270. 7397213 10.1016/0005-2795(80)90254-8

[38] Burgoyne RD, Weiss JL (2001) The neuronal calcium sensor family of Ca^2+^-binding proteins. Biochem J 353: 1–12. 11115393 PMC1221537

[39] Burgoyne RD (2007) Neuronal calcium sensor proteins: generating diversity in neuronal Ca^2+^ signalling. Nat Rev Neurosci 8: 182–193. 17311005 10.1038/nrn2093 PMC1887812

[40] Ames JB, Lim S (2012) Molecular structure and target recognition of neuronal calcium sensor proteins. Biochim Biophys Acta 1820: 1205–1213. 22020049 10.1016/j.bbagen.2011.10.003 PMC3266469

[41] Pongs O, Lindemeier J, Zhu XR, Theil T, Engelkamp D, et al (1993) Frequenin-a novel calcium-binding protein that modulates synaptic efficacy in the *Drosophila* nervous system. Neuron 11: 15–28. 8101711 10.1016/0896-6273(93)90267-u

[42] McCue HV, Haynes LP, Burgoyne RD (2010) The diversity of calcium sensor proteins in the regulation of neuronal function. Cold Spring Harbor Perspectives in Biology 2: a004085. 20668007 10.1101/cshperspect.a004085 PMC2908765

[43] De Castro E, Nef S, Fiumelli H, Lenz SE, Kawamura S, et al (1995) Regulation of rhodopsin phosphorylation by a family of neuronal calcium sensors. Biochem Biophys Res Comm 216: 133–140. 7488079 10.1006/bbrc.1995.2601

[44] Putnam NH, Srivastava M, Hellsten U, Dirks B, Chapman J, et al (2007) Sea anemone genome reveals ancestral eumetazoan gene repertoire and genomic organization. Science 317: 86–94. 17615350 10.1126/science.1139158

[45] Zoccola D, Tambutte E, Kulhanek E, Puverel S, Scimeca JC, et al (2004) Molecular cloning and localization of a PMCA P-type calcium ATPase from the coral *Stylophora pistillata* . Biochim Biophys Acta 1663: 117–126. 15157614 10.1016/j.bbamem.2004.02.010

[46] Ishida H, Nakashima K, Kumaki Y, Nakata M, Hikichi K, et al (2002) The solution structure of apocalmodulin from *Saccharomyces cerevisiae* implies a mechanism for its unique Ca^2+^ binding property. Biochem 41: 15536–15542. 12501182 10.1021/bi020330r

[47] O’Donnell SE, Newman RA, Witt TJ, Hultman R, Froehlig JR, et al (2009) Thermodynamics and conformational change governing domain-domain interactions of calmodulin. Methods Enzymol 466: 503–526. 21609874 10.1016/S0076-6879(09)66021-3

[48] O’Donnell SE, Yu L, Fowler CA, Shea MA (2011) Recognition of beta-calcineurin by the domains of calmodulin: thermodynamic and structural evidence for distinct roles. Proteins 79: 765–786. 21287611 10.1002/prot.22917 PMC3057930

[49] Amici M, Doherty A, Jo J, Jane D, Cho K, et al (2009) Neuronal calcium sensors and synaptic plasticity. Biochem Soc Trans 37: 1359–1363. 19909276 10.1042/BST0371359

[50] Hilfiker S (2003) Neuronal calcium sensor-1: a multifunctional regulator of secretion. Biochem Soc Trans 31: 828–832. 12887315 10.1042/bst0310828

[51] Weiss JL, Hui H, Burgoyne RD (2010) Neuronal calcium sensor-1 regulation of calcium channels, secretion, and neuronal outgrowth. Cell Mol Neurobiol 30: 1283–1292. 21104311 10.1007/s10571-010-9588-7 PMC11498851

[52] Haynes LP, Fitzgerald DJ, Wareing B, O’Callaghan DW, Morgan A, et al (2006) Analysis of the interacting partners of the neuronal calcium-binding proteins L-CaBP1, hippocalcin, NCS-1 and neurocalcin delta. Proteomics 6: 1822–1832. 16470652 10.1002/pmic.200500489

[53] Ivings L, Pennington SR, Jenkins R, Weiss JL, Burgoyne RD (2002) Identification of Ca^2+^-dependent binding partners for the neuronal calcium sensor protein neuroncalcin: interaction with actin, clathrin and tubulin. Biochem J 363: 599–608. 11964161 10.1042/0264-6021:3630599 PMC1222513

[54] Clode PL, Marshall AT (2004) Calcium localisation by X-ray microanalysis and fluorescence microscopy in larvae of zooxanthellate and azooxanthellate corals. Tissue Cell 36: 379–390. 15533453 10.1016/j.tice.2004.06.005

[55] Vandermeulen JH, Watabe N (1973) Studies on Reef Corals. I. Skeleton formation by newly settled planula larva of *Pocillopora damicornis* . Mar Biol 23: 47–57.

[56] Vandermeulen JH (1974) Studies on Reefs Corals II. Fine structure of planktonic planula larva of *Pocillopora damicornis* with emphasis on the aboral epidermis. Mar Biol 27: 239–249.

